# Advanced diffusion imaging sequences could aid assessing patients with focal cortical dysplasia and epilepsy^☆^

**DOI:** 10.1016/j.eplepsyres.2013.11.004

**Published:** 2014-02

**Authors:** Gavin P. Winston, Caroline Micallef, Mark R. Symms, Daniel C. Alexander, John S. Duncan, Hui Zhang

**Affiliations:** a Epilepsy Society MRI Unit, Department of Clinical and Experimental Epilepsy, UCL Institute of Neurology, Queen Square, London WC1N 3BG, United Kingdom; b Lysholm Department of Neuroradiology, National Hospital for Neurology and Neurosurgery, University College London Hospitals NHS Foundation Trust, Queen Square, London WC1N 3BG, United Kingdom; c Department of Computer Science & Centre for Medical Image Computing, University College London, Gower Street, London WC1E 6BT, United Kingdom

**Keywords:** CNR, contrast-to-noise ratio, CSF, cerebrospinal fluid, FA, fractional anisotropy, FCD, focal cortical dysplasia, FLAIR, fluid-attenuated inversion recovery, ICVF, intracellular volume fraction, ITG, inferior temporal gyrus, MCD, malformations of cortical development, MD, mean diffusivity, MFG, middle frontal gyrus, MRI, magnetic resonance imaging, NODDI, neurite orientation dispersion and density imaging, ODI, orientation dispersion index, PROPELLER, periodically rotated overlapping parallel lines with enhanced reconstruction, Diffusion imaging, Focal cortical dysplasia, Epilepsy surgery, NODDI, Neurite density

## Abstract

Malformations of cortical development (MCD), particularly focal cortical dysplasia (FCD), are a common cause of refractory epilepsy but are often invisible on structural imaging. NODDI (neurite orientation dispersion and density imaging) is an advanced diffusion imaging technique that provides additional information on tissue microstructure, including intracellular volume fraction (ICVF), a marker of neurite density.

We applied this technique in 5 patients with suspected dysplasia to show that the additional parameters are compatible with the underlying disrupted tissue microstructure and could assist in the identification of the affected area.

The consistent finding was reduced ICVF in the area of dysplasia. In one patient, an area of reduced ICVF and increased fibre dispersion was identified that was not originally seen on the structural imaging. The focal reduction in ICVF on imaging is compatible with previous iontophoretic data in surgical specimens, was more conspicuous than on other clinical or diffusion images (supported by an increased contrast-to-noise ratio) and more localised than on previous DTI studies.

NODDI may therefore assist the clinical identification and localisation of FCD in patients with epilepsy. Future studies will assess this technique in a larger cohort including MRI negative patients.

## Highlights

• Malformations of cortical development are a common cause of refractory epilepsy.
• They are often invisible on structural imaging and only detected following surgery.
• We assess a novel diffusion imaging technique (NODDI) in patients with dysplasia.
• This shows more conspicuous changes than other clinical or diffusion scans.
• This technique may assist the identification of FCD in patients with epilepsy.

## Introduction

A third of patients with focal epilepsy are refractory to medical treatment. Identification of the epileptogenic zone is critical in planning surgical treatment but up to 20–30% of patients have normal structural magnetic resonance imaging (MRI) scans (Duncan, 2010). Drug-resistant epilepsy is associated with malformations of cortical development (MCD) in 15–20% of adult patients and over 50% of paediatric patients.

The most common type, focal cortical dysplasia (FCD), is frequently not detected on structural MRI and up to 42% of MRI-negative patients undergoing surgery have FCD (Chapman et al., 2005). FCD is characterised by disrupted laminar architecture and columnar organisation and abnormal cells, including dysmorphic neurons and balloon cells (Blumcke et al., 2011). Studies on neocortical tissue from surgically resected temporal lobe specimens with FCD demonstrate altered diffusion parameters in the extracellular space and, in type II, a reduced intracellular volume fraction (ICVF) (Vargova et al., 2011).

Typical neuroimaging features of FCD including cortical thickening and blurring of the grey-white matter boundary on T1-weighted images and cortical and subcortical signal hyperintensities on T2-weighted images are not always present (Barkovich and Kuzniecky, 1996). Diffusion tensor imaging (DTI) demonstrates abnormal diffusion indices in underlying white matter, including reduced fractional anisotropy (FA) and increased mean diffusivity (MD). However these are non-specific, extend beyond the area of abnormality (Eriksson et al., 2001) and cannot evaluate dysplastic grey matter due to the low FA and signal contamination by cerebrospinal fluid (CSF).

The assumption inherent in DTI that each voxel contains a single tissue compartment with Gaussian diffusion is increasingly recognised as inadequate. Multi-compartment models more accurately reflect the diffusion MR signal by modelling several tissue compartments and distinguishing restricted non-Gaussian diffusion (intracellular) from hindered Gaussian diffusion (extracellular space) but the lengthy scans required are often clinically unfeasible (Panagiotaki et al., 2012).

The NODDI (neurite orientation dispersion and density imaging) model includes three compartments for each voxel – intracellular, extracellular and CSF – and provides additional estimates of tissue microstructure in both grey and white matter. It distinguishes two key variables contributing to changes in FA – neurite density (ICVF) and fibre orientation dispersion – with a clinically feasible scan protocol of 20 min (Zhang et al., 2012) so could potentially assist the identification and understanding of FCD.

We describe a preliminary study in which the NODDI model is applied for the first time in a clinical population of patients with epilepsy and suspected dysplasia on conventional imaging. The aims are to determine firstly whether the parameters estimates are compatible with the underlying disrupted tissue microstructure and secondly whether they potentially provide useful additional clinical information for localising the abnormality. This proof-of-concept study lays the foundation for future larger studies.

## Methods

Five consecutive patients with previous structural imaging findings compatible with FCD (4 patients) or tuberous sclerosis (TS, 1 patient) attending for further imaging as part of pre-surgical assessment were invited to undergo an additional NODDI protocol optimised for a 3T GE Signa HDx scanner (Alexander, 2008). The study was approved by the National Hospital for Neurology and Neurosurgery and the Institute of Neurology Joint Research Ethics Committee, and informed written consent was obtained from all subjects. Patient demographics and clinical data are listed in Table 1.

The protocol consisted of two high angular resolution diffusion imaging shells (single-shot EPI, 50 mm × 2.5 mm axial slices, 96 × 96 matrix zero-filled to 128 × 128, field-of-view 24 cm × 24 cm, TE 85 ms, TR 13 s, 9 non-diffusion weighted acquisitions, 24 directions with *b*-value 700 s/mm^2^, 48 directions with *b*-value 2000 s/mm^2^, maximum gradient strength 40 mT/m, slew rate 150 T/m/s, total scan time 20 min). Optimised gradient directions from the Camino software package generated using electrostatic energy minimisation were used (Cook et al., 2007).

Fitting was performed with the NODDI Matlab Toolbox http://www.nitrc.org/projects/noddi_toolbox to generate maps of ICVF and orientation dispersion index (ODI). ICVF estimates the intracellular volume as a fraction of the non-CSF compartment as a marker of neurite density, whilst ODI provides an index of the degree of dispersion of the fibre orientations ranging from 0 (no dispersion) to 1 (fully dispersed). A mask comprising all brain voxels with a CSF fraction below 90% was applied to the maps as fitting of ICVF and ODI may be inaccurate in voxels that are predominantly CSF.

In view of the small sample size, the parametric maps were first qualitatively compared to abnormalities in the structural images and maps of standard DTI measures (FA and MD). In addition, the contrast-to-noise ratio was determined for the diffusion scans using the following formula (Song et al., 2004):

$$\text{CNR}=\frac{\left|S_{\text{lesion}}-S_{\text{contralateral}}\right|}{\sqrt{[{\left(SD_{\text{lesion}}\right)}^{2}+{\left(SD_{\text{contralateral}}\right)}^{2}]/2}}$$

where *S*_lesion_ and *S*_contralateral_ represent the mean values in the lesion and homologous contralateral cortex and *SD*_lesion_ and *SD*_contralateral_ are the standard deviations in these regions of interest.

## Results

**Fig. 1:**
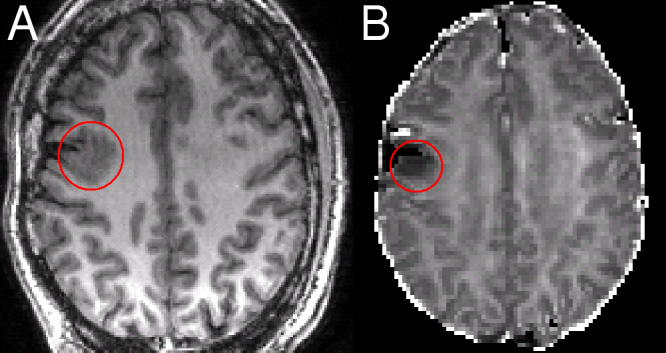
Residual FCD following a right middle frontal gyrus resection. Patient 1: the residual FCD on the T1-weighted image (A) corresponds with an area of reduced ICVF (B).

**Fig. 2:**
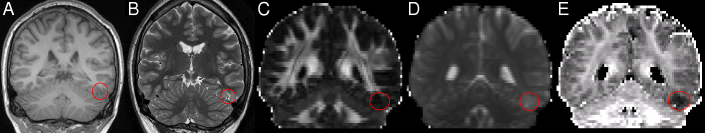
Left inferior temporal gyrus FCD. Patient 2: the FCD is poorly defined on structural images including volumetric T1-weighted (A) and T2-weighted PROPELLER (B) and standard DTI images including FA (C) and MD (D) but easily visible as reduced ICVF (E).

In three patients with suspected FCD (patients 1–3) and the patient with TS (patient 4), areas of reduced ICVF were clearly identified that co-located with the abnormality (Figs. 1 and 2). No change was apparent in the ODI. Of particular note, the change in ICVF was more conspicuous than on conventional structural or diffusion images (Fig. 2).

In the fifth patient, the structural imaging was initially felt to be normal but on review a malformation of cortical development, most likely FCD or possibly polymicrogyria, was detected. The abnormal area was clearly evident on NODDI imaging, with reduced ICVF and increased ODI (Supplementary Figure 1, fifth row).

The mean contrast-to-noise ratio (CNR) for the ICVF across all subjects was 3.60 (standard deviation 1.73). This was significantly greater than the CNR for FA (mean 1.21, standard deviation 0.45; two-tailed paired *t*-test *p* = 0.041) and showed a trend to being greater than the CNR for MD (mean 2.80, standard deviation 1.34; two-tailed paired *t*-test *p* = 0.080).

A comparison of structural and diffusion imaging data for all five patients is presented in Supplementary Figure 1.

## Discussion

The additional microstructural information readily identified the area of abnormality in all patients. The consistent change was a localised fall in the ICVF that is compatible with previous iontophoretic data (Vargova et al., 2011), more readily identifiable than on other clinical or diffusion images with a higher CNR than FA or MD images and more localised than previous diffusion studies employing FA and MD. The tubers in tuberous sclerosis are histologically comparable to FCD type IIb and the differential diagnosis of polymicrogyria (in patient 5) is also associated with a reduced neuronal density (Judkins et al., 2011) that manifests as reduced ICVF.

DTI indices such as FA reflect many underlying parameters including neuronal density, fibre orientation dispersion, axonal diameter and degree of myelination and are thus non-specific. The NODDI model disentangles the different factors contributing to the change in FA. In particular, it separates the influence of neurite density and orientation dispersion into distinct indices. The technique is suitable for both grey and white matter. Moreover, by modelling CSF as a separate compartment, it avoids CSF contamination, a further confound on traditional indices such as FA.

Previous studies have suggested that in FCD the reduced FA may result from increased or abnormally located grey matter or pathological white matter with abnormal myelination or ectopic neurones, whilst the increase in MD may result from defective neurogenesis or cell loss resulting in increased extracellular space (Eriksson et al., 2001). In this small series our results support the interpretation that diffusion changes are in part due to an increase in extracellular space.

This study presents preliminary data that needs confirmation in a larger cohort and comparison with post-surgical histology. In particular, we included predominantly patients with abnormalities detected on conventional imaging to establish the validity of the technique. This may now be usefully extended to larger number of patients with malformations and acquired cerebral lesions to characterise the range of findings and then to those individuals with no discernible abnormality on conventional brain imaging. As the data is inherently quantitative, a larger cohort would enable voxel-based quantitative comparisons with a group of healthy controls as has previously been performed on FLAIR images in MRI-negative patients (Focke et al., 2009).

The main limitation is the relatively large voxel size of diffusion imaging (here 1.875 mm × 1.875 mm × 2.5 mm) in comparison to what can be obtained with structural imaging (typically 1 mm isotropic) although grey and white matter can still be resolved. Reducing voxel size whilst maintaining a clinically feasible scan duration is possible only with the stronger imaging gradients available on modern scanners (50–60 mT/m).

In conclusion, we have shown that NODDI is viable to apply to a clinical population and the findings of reduced ICVF are compatible with the known pathology of FCD. NODDI may assist the clinical identification of FCD in patients with epilepsy that is not easily seen on other imaging sequences and requires further study. The sequence can be readily implemented on MRI scanners from all manufacturers.

## Funding source

Gavin Winston was supported by a Clinical Research Training Fellowship from the Medical Research Council (G0802012). The Engineering and Physical Sciences Research Council (grant EP/E007748) and the EU CONNECT consortium supported the contributions of Daniel Alexander and Hui Zhang. We are grateful to the Wolfson Trust and the Epilepsy Society for supporting the Epilepsy Society MRI scanner. This work was supported by the National Institute for Health Research University College London Hospitals Biomedical Research Centre.

No sponsor had any role in the study design; in the collection, analysis and interpretation of data; in the writing of the report; and in the decision to submit the article for publication.

## Disclosure of conflicts of interest

Prof. Duncan has served as a consultant for GE Healthcare on the development of PET tracers. The remaining authors have no conflicts of interest.

## Figures and Tables

**Figure:**
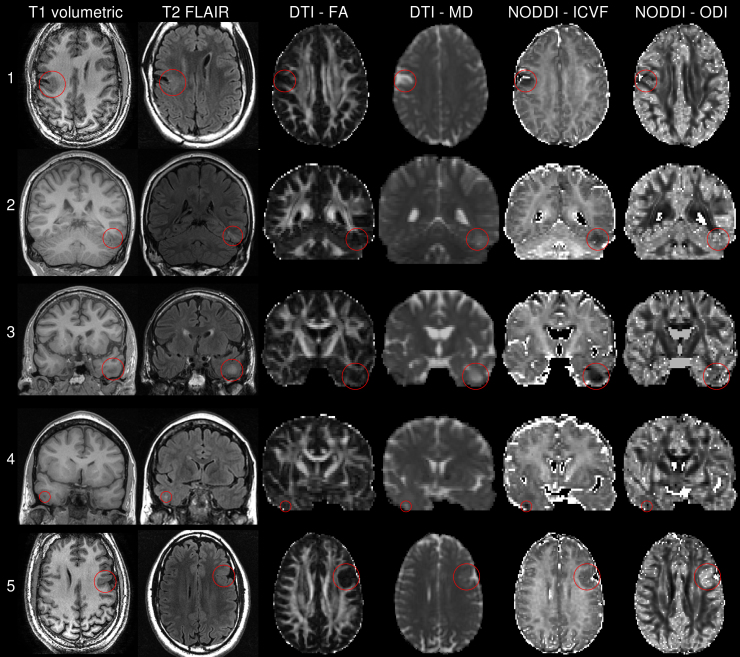


**Table 1:** Demographic and clinical characteristics of patients.

Patient	Age/gender	Age at seizure onset (years)	Structural MRI report	Video EEG localisation
1	21/M	2	Right MFG resection with residual FCD	Right frontocentral
2	27/M	8	Left ITG FCD	Left anterior temporal
3	62/M	17	Left ITG FCD/dysplasia	Left anterior temporal
4	31/F	6	Cortical tubers, largest in right ITG	Right anterior temporal
5	28/M	10	Normal, then L MFG MCD (FCD or polymicrogyria)	Left frontocentral
